# Engineering of Long-Circulating Peptidoglycan Hydrolases Enables Efficient Treatment of Systemic Staphylococcus aureus Infection

**DOI:** 10.1128/mBio.01781-20

**Published:** 2020-09-22

**Authors:** Anna M. Sobieraj, Markus Huemer, Léa V. Zinsli, Susanne Meile, Anja P. Keller, Christian Röhrig, Fritz Eichenseher, Yang Shen, Annelies S. Zinkernagel, Martin J. Loessner, Mathias Schmelcher

**Affiliations:** a Institute of Food, Nutrition and Health, ETH Zurich, Zurich, Switzerland; b Department of Infectious Diseases and Hospital Epidemiology, University Hospital Zurich, University of Zurich, Zurich, Switzerland; Institut Pasteur

**Keywords:** endolysin, protein therapeutic, antibiotic resistance, MRSA, circulation half-life

## Abstract

Staphylococcus aureus is a human pathogen causing life-threatening diseases. The increasing prevalence of multidrug-resistant S. aureus infections is a global health concern, requiring development of novel therapeutic options. Peptidoglycan-degrading enzymes (peptidoglycan hydrolases, PGHs) have emerged as a highly effective class of antimicrobial proteins against S. aureus and other pathogens. When applied to Gram-positive bacteria, PGHs hydrolyze bonds within the peptidoglycan layer, leading to rapid bacterial death by lysis. This activity is highly specific and independent of the metabolic activity of the cell or its antibiotic resistance patterns. However, systemic application of PGHs is limited by their often low activity *in vivo* and by an insufficient serum circulation half-life. To address this problem, we aimed to extend the half-life of PGHs selected for high activity against S. aureus in human serum. Half-life extension and increased serum circulation were achieved through fusion of PGHs to an albumin-binding domain (ABD), resulting in high-affinity recruitment of human serum albumin and formation of large protein complexes. Importantly, the ABD-fused PGHs maintained high killing activity against multiple drug-resistant S. aureus strains, as determined by *ex vivo* testing in human blood. The top candidate, termed ABD_M23, was tested *in vivo* to treat S. aureus-induced murine bacteremia. Our findings demonstrate a significantly higher efficacy of ABD_M23 than of the parental M23 enzyme. We conclude that fusion with ABD represents a powerful approach for half-life extension of PGHs, expanding the therapeutic potential of these enzybiotics for treatment of multidrug-resistant bacterial infections.

## Abstract (precis)

Life-threatening infections with Staphylococcus aureus are often difficult to treat due to the increasing prevalence of antibiotic-resistant bacteria and their ability to persist in protected niches in the body. Bacteriolytic enzymes are promising new antimicrobials because they rapidly kill bacteria, including drug-resistant and persisting cells, by destroying their cell wall. However, when injected into the bloodstream, these enzymes are not retained long enough to clear an infection. Here, we describe a modification to increase blood circulation time of the enzymes and enhance treatment efficacy against S. aureus-induced bloodstream infections. This was achieved by preselecting enzyme candidates for high activity in human blood and coupling them to serum albumin, thereby preventing their elimination by kidney filtration and blood vessel cells.

## INTRODUCTION

Staphylococcus aureus is an opportunistic pathogen that can cause serious disease in susceptible individuals. Immunocompromised patients, such as people with chronic illnesses (e.g., diabetes and cancer), surgical patients, the elderly, or prosthetic device carriers are under an increased risk of infection (1). While some conditions caused by S. aureus are superficial and rather mild (e.g., impetigo, folliculitis, and cutaneous abscesses), others (e.g., endocarditis, osteomyelitis, meningitis, toxic shock syndrome, bacteremia, and sepsis) are life-threatening (1, 2). In fact, S. aureus bacteremia is associated with mortality rates of up to 67% (3). The difficulty to treat these infections is based on several factors, such as the ability of S. aureus to form biofilms (4) and abscesses (5), the capability of invading and persisting in human cells (6), and the large repertoire of resistance mechanisms against conventional antibiotics (7), as well as the lack of development of novel antimicrobials (8). The prevalence of methicillin-resistant S. aureus (MRSA) is a major global health care concern (9, 10) and a severe economic burden (11). For these reasons, developing novel classes of antibacterial agents is paramount (12).

Peptidoglycan hydrolases (PGHs) are peptidoglycan-degrading enzymes that constitute a novel class of antimicrobials. Bacteriophages (viruses infecting bacteria) use PGHs termed endolysins to destroy the bacterial cell wall at the end of their lytic replication cycle in order to release progeny (13, 14). Other bacteriophage-encoded PGHs include virion-associated lytic proteins required for DNA entry into the host bacterium (15). PGHs of bacterial origin include autolysins (16) and a few bacteriocins (17). Despite their diverse origins, the basic function of PGHs remains the same, i.e., hydrolysis of the bacterial cell wall. In Gram-positive bacteria, PGHs can be applied externally to rapidly hydrolyze the peptidoglycan and cause bacterial lysis. Unlike most antibiotic drugs, PGHs are not dependent on an active bacterial metabolism and are highly specific for the target organism on the genus and even species levels, which avoids selection pressure on commensal populations (13, 18). Endolysins and some bacteriocins such as lysostaphin (LST) (19) and ALE-1 (20) harbor a modular architecture, in which cell wall binding and catalytic functions are localized on different but linked domains (21–25). Such a modular structure enables molecular engineering by adding and swapping domains of different origins, producing chimeric enzymes with novel functions (reviewed in reference 26). The *in vivo* therapeutic potential of PGHs has been successfully demonstrated (27–34), and the encouraging results have led to evaluation of PGHs in human clinical trials (reviewed in reference 35).

Besides their intrinsic advantages, there are also limitations for application of PGHs as potential therapeutics, especially in the treatment of systemic infections. PGHs, due to their proteinaceous nature, generally feature a relatively short serum circulation half-life. For example, the half-life of lysostaphin was previously found to be 1 h in mice (36) and 1.5 h in rats (37), that of Cpl-1 was found to be 20.5 min in mice (38), and that of SAL200 was found to be 0.3 to 9.7 h in monkeys (39) and 2.4 to 22.8 min in humans (40). The longest half-life of 23 h was reported for an engineered version of LysK, CSL[Eu], in mice (41). Elimination of proteins from the organism occurs through a combination of processes, which include general proteolysis, endocytosis and lysosomal degradation, renal filtration, and hepatic elimination (42). Elimination through renal filtration could be controlled by increasing the size of proteins above 70 kDa, which is reported to be the molecular weight cutoff value of the renal filters (43), e.g., by PEGylation (36, 44) or dimerization (38, 45). Another strategy that aims to bypass both endocytosis followed by lysosomal degradation and renal filtration involves fusion to serum proteins, which feature a long half-life due to the recycling pathway operating via the neonatal Fc receptor (FcRn) (46). An example of such an approach is fusion of an albumin-binding domain (ABD) to the protein of interest. ABD is a 5.6-kDa domain originating from the streptococcal protein G and binding human serum albumin (HSA) with high affinity (47, 48). Proteins covalently fused to ABD can form a high-affinity complex with HSA, which is then rescued from renal filtration due to its large hydrodynamic volume and is protected from lysosomal degradation by FcRn recycling (49). For LysK (41) and lysostaphin (37), this resulted in significant extension of their half-lives (37, 41) and in other improvements in pharmacodynamics (37). While these results are encouraging, no studies have shown that half-life extension of PGHs translates into enhanced efficacy in infection models. Additionally, all previous studies used PGHs that had not been screened or selected for optimal properties to treat systemic infections.

Here, we used extensive prescreening under relevant conditions to select the best candidate enzyme constructs for engineering of enzybiotics featuring increased serum half-life via ABD fusion. Furthermore, we demonstrate successful application in a murine model of systemic S. aureus infection.

## RESULTS

### Selection of PGHs with high activity in human serum.

**FIG 1:**
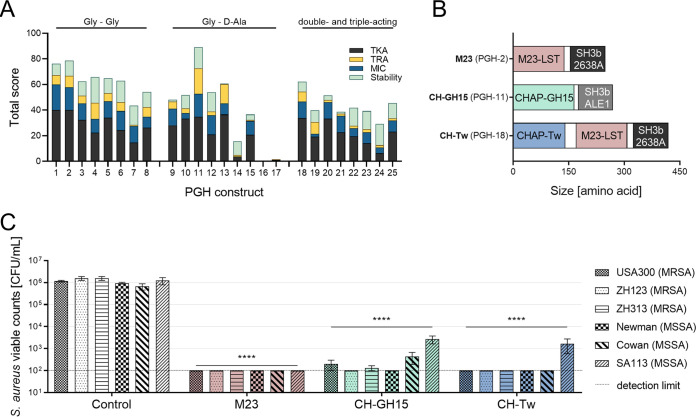
Outline of screening and selection of PGHs with best activity against S. aureus in human serum. (A) Characterization of 25 most promising PGH candidates by time-kill assays (TKA), turbidity reduction assays (TRA), MIC assays, and stability assays. Enzymes were grouped according to their enzymatic specificity into glycine-glycine endopeptidases (Gly – Gly), glycine–d-alanine endopeptidases (Gly – d-Ala), and enzymes harboring catalytic domains with various specificities (double- and triple-acting). The highest-scoring candidate from each group was selected for further experiments. (B) Schematic representation of the selected PGHs, including their sizes and domain homologies. (C) Activity of the selected PGHs M23, CH-GH15, and CH-Tw against MSSA and MRSA strains. Viable bacterial counts were determined after 10 min of incubation with 100 nM PGHs in human serum. Error bars indicate standard errors of the means of results from three biological replicates. The dashed line corresponds to the detection limit of 100 CFU/ml. Bacterial counts below the detection limit are displayed as 100 CFU/ml. Asterisks indicate significant difference from the control (****, *P* < 0.0001).

To identify PGH constructs featuring high antibacterial activity against S. aureus in human serum, we first modified a previously established protocol (50) to screen a comprehensive collection of more than 300 parental and engineered staphylococcal PGHs. This procedure has previously been successfully applied to identify lytic enzymes highly active in milk (50) and under intracellular conditions (51) and was adapted here for human serum. Applying this protocol in an iterative fashion, using S. aureus Newman as a target strain, enabled us to narrow down the selection of possible candidates to the 25 most promising constructs (PGH-1 to PGH-25; see Table S1 in the supplemental material). These included enzymes with domains originating from the bacteriocins lysostaphin (LST) (19) and ALE-1 (20) and lytic enzymes from bacteriophages such as 2638A (52), Twort (53), K (54), H5 (55), 11 (56), 187 (57), and GH15 (30). Of note, this selection contained representatives of three mechanistic groups, i.e., glycine-glycine endopeptidases (Gly-Gly), which cleave within the pentaglycine bridge of S. aureus peptidoglycan; glycine–d-alanine endopeptidases (Gly–d-Ala), which cut the peptidoglycan between the pentaglycine bridge and the stem peptide; and enzymes harboring two or three catalytic domains of different enzymatic activity (double-/triple-acting). The 25 selected PGHs were expressed and purified and underwent comprehensive characterization *in vitro* to compare their levels of killing efficacy (time-kill assay, TKA) and growth inhibition (MIC) and their cell lysis kinetics (turbidity reduction assay, TRA) against S. aureus and their levels of stability in human serum (Fig. 1A; see also Table S2). This comparative analysis allowed selection of the top-scoring enzyme from each group. These were M23LST(L)_SH3b2638A (M23; PGH-2), CHAPGH15_SH3bALE1 (CH-GH15; PGH-11), and CHAPTw_M23LST(L)_SH3b2638A (CH-Tw; PGH-18) from the Gly-Gly, Gly–d-Ala, and double-/triple-acting group, respectively (Fig. 1B). Activity of the selected enzymes was confirmed by TKAs in human serum using multiple S. aureus strains, including MRSA and methicillin-susceptible S. aureus (MSSA) (Fig. 1C). All enzymes reduced the bacterial load very significantly (*P* < 0.0001) and, in most cases, to nondetectable numbers.

10.1128/mBio.01781-20.4 TABLE S1 List of peptidoglycan hydrolases (PGHs) used in the study. PGH-1 to PGH-25 were part of the laboratory collection. ABD-fused PGHs were constructed in this study. Download Table S1, PDF file, 0.5 MB. Copyright © 2020 Sobieraj et al. 2020 Sobieraj et al. This content is distributed under the terms of the Creative Commons Attribution 4.0 International license.

10.1128/mBio.01781-20.5 TABLE S2 Characterization of PGHs identified in the screening by time-kill assay (TKA), turbidity reduction assay (TRA), MIC assay, and stability assay (SA). Results of each assay were normalized to the highest scorer. The final score was calculated based on a weighted sum as follows: 4×TKA + 2×MIC + 2×TRA + 2×SA. Enzymes were grouped into three categories based on their cleavage specificity as follows: (i) Gly-Gly endopeptidases; (ii) Gly–d-Ala endopeptidases; (iii) double-acting and triple-acting enzymes. The highest-scoring enzyme from each category (shown in bold) was selected for further experiments. ND, not determined. Download Table S2, PDF file, 0.5 MB. Copyright © 2020 Sobieraj et al. 2020 Sobieraj et al. This content is distributed under the terms of the Creative Commons Attribution 4.0 International license.

### Activity of ABD-fused PGHs depends on the position of the ABD within the enzyme.

**FIG 2:**
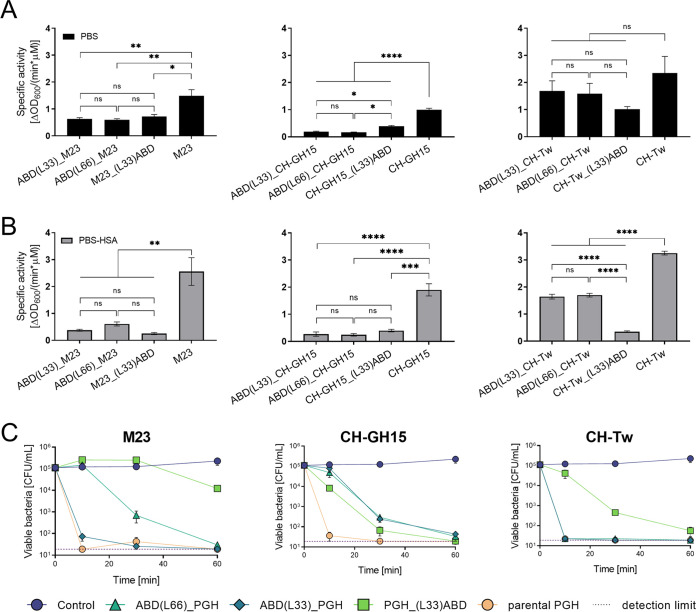
*In vitro* activity of parental and ABD-fused PGH variants. (A and B) Turbidity reduction assays were conducted in PBS (A) and in PBS supplemented with human serum albumin (B). (C) Time-kill assays comparing the bactericidal activities of parental and ABD-fused PGH variants at a concentration of 100 nM in human serum. Error bars indicate standard errors of the means of results from three biological replicates. The dashed line corresponds to the detection limit of 20 CFU/ml. Asterisks indicate statistical significance (*, *P* < 0.05; **, *P* < 0.01; ***, *P* < 0.001; ****, *P* < 0.0001; ns, nonsignificant, *P* > 0.05).

With the aim of extending the serum circulation half-life of the top three candidate PGHs M23, CH-GH15 and CH-Tw, we inserted an ABD at various positions within the polypeptides and tested different linkers connecting the individual domains. Enzymatic activity of the resulting fusions in comparison with the respective parental PGHs was tested by TRAs in phosphate-buffered saline (PBS) (Fig. 2A) and PBS supplemented with 5 μM human serum albumin (PBS-HSA) (Fig. 2B), and TKAs in human serum (Fig. 2C). In most cases, modification with an ABD significantly reduced the activity off the enzymes in TRAs. Parental PGH activities were increased by adding HSA, as previously observed for other staphylolytic PGHs (41, 58) (Fig. 2A and B). In contrast, the activities of the respective ABD variants did not change or decreased slightly when HSA was added (Fig. 2B). In TKAs, however, the differences in activity between parental and ABD-fused enzymes were less pronounced. Interestingly, the most active ABD variants of M23 and CH-Tw {ABD(L33)_M23 [where “L33” represents a flexible 33-amino-acid (aa) linker] [ABD_M23] and ABD(L66)_CH-Tw [ABD_CH-Tw], respectively} exhibited levels of activity similar to those exhibited by the parental enzymes (Fig. 2C). Despite similar activity levels of ABD(L33)_CH-Tw and ABD(L66)_CH-Tw in both assays, the latter turned out to be easier to purify and achieved higher yields (data not shown). Therefore, the ABD fusion constructs ABD(L33)_M23 (ABD_M23), CH-GH15_(L33)ABD (CH-GH15_ABD), and ABD(L66)_CH-Tw (ABD_CH-Tw) were selected for further experiments.

### PGHs fused to ABD bind human serum albumin with high affinity.

**FIG 3:**
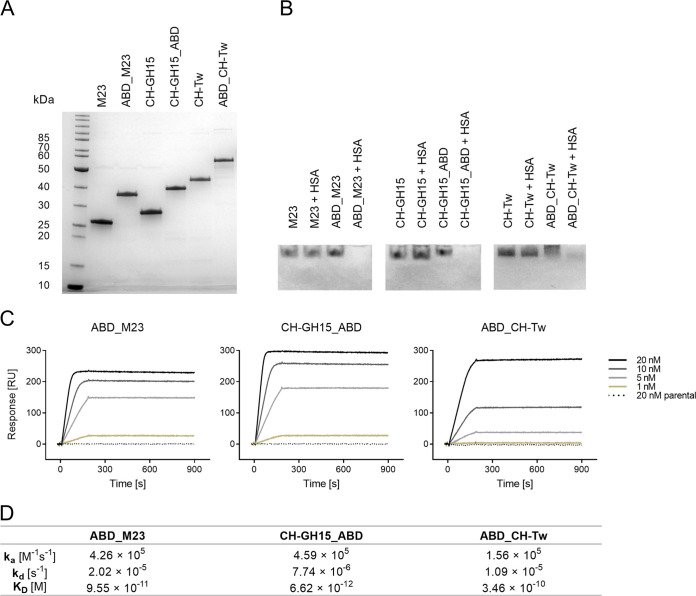
Binding of albumin-binding domain (ABD)-fused peptidoglycan hydrolases to human serum albumin. (A) SDS-PAGE analysis of the parental PGHs and the corresponding selected ABD-fusion constructs. (B) Electrophoretic mobility shift assay with parental and ABD-fused PGH constructs that had or had not been incubated with an excess of HSA. Proteins forming a complex with HSA displayed a shift in migration. (C) Surface plasmon resonance (SPR) kinetic analysis of ABD-fused PGH constructs at concentrations ranging from 1 nM to 20 nM interacting with HSA (immobilized). Association was measured for 180 s and dissociation for 720 s at a constant flow rate of 10 μl/min. Injections of parental enzymes (20 nM) served as controls. RU, resonance units. (D) Quantification of the interaction between ABD-fused PGHs and HSA by SPR. A fitting to the mass-transfer model was performed for both association and dissociation phases to determine the rate and equilibrium affinity constants. k_a_, association rate constant; k_d_, dissociation rate constant; K_D_, equilibrium dissociation constant.

To investigate whether the ABD remains functional when fused to the selected PGHs, we tested binding of parental and ABD-fused PGHs (Fig. 3A) to HSA in an electrophoretic mobility shift assay (EMSA) (Fig. 3B). While all of the parental PGHs (irrespective of HSA incubation) and ABD-fused PGHs without previous HSA incubation migrated toward the cathode due to their high isoelectric points (pI > 9.5), ABD-fused PGHs that had been incubated with HSA failed to migrate into the gel. This was presumably due to the formation of a high-molecular-mass complex between HSA (pI = 4.9 [59]) and the PGHs with a net pI lower than 8.3 (which is the pH of the running buffer used in the EMSA). Further evidence that ABD_M23, CH-GH15_ABD, and ABD_CH-Tw bind to HSA was provided by surface plasmon resonance (SPR) analysis, in which the real-time interactions between immobilized HSA and parental or ABD-fused PGHs in solution could be measured (Fig. 3C). Consistent with the EMSA results, no interaction of parental PGHs with HSA was observed, whereas ABD_M23, CH-GH15_ABD, and ABD_CH-Tw bound HSA with high affinity (Fig. 3D).

### ABD fusion constructs retain high enzymatic activity against S. aureus in whole human blood and are not cytotoxic.

**FIG 4:**
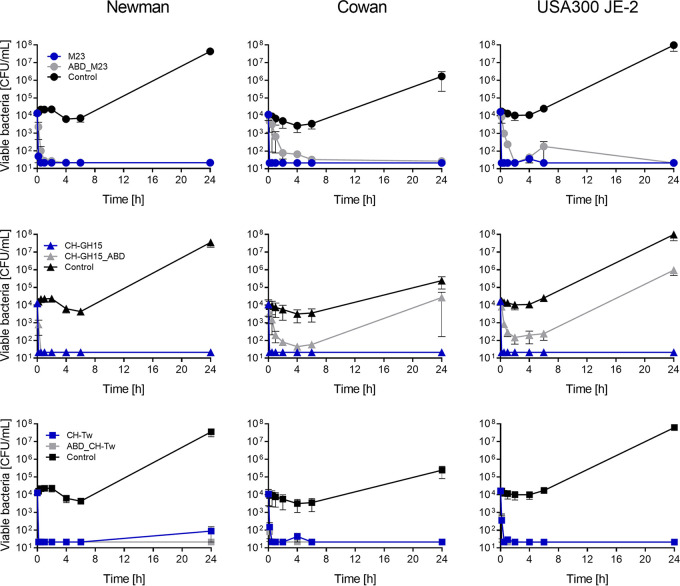
*Ex vivo* activity of parental and ABD-fused PGH constructs against S. aureus in whole human blood. Fresh blood spiked with S. aureus Newman, Cowan, or USA300 JE-2 was treated with 100 nM PGH. Viable bacterial counts were monitored for 24 h of incubation at 37°C. Error bars indicate standard errors of the means of results from at least three biological replicates. The detection limit was 21.2 CFU/ml.

To further evaluate the potential of our enzymes as therapeutics for the treatment of blood-borne S. aureus infections, we determined the activity of parental PGHs and ABD fusion constructs *ex vivo* in whole human blood against three S. aureus strains (Fig. 4). The highest activities were observed for the parental PGHs and the ABD_CH-Tw construct, which reduced viable S. aureus counts below detectable levels (3 log units) and maintained those levels after the first 10 to 30 min of treatment for all tested strains. ABD_M23 displayed high activity against all tested strains, with no detectable bacteria after 24 h. All enzymes showed high activity against S. aureus Newman, reducing its viable counts by 3 log units within the first hour of treatment. Additionally, we tested the cytotoxicity of both parental and ABD-fused PGHs to human MG-63 cells, and no effects were observed (see Fig. S1 in the supplemental material).

10.1128/mBio.01781-20.1 FIG S1 Cytotoxicity of parental and ABD-fused PGHs in MG-63 cells after 22 h. Cells were incubated with parental or ABD-fused constructs for 22 h, and LDH levels were measured in the supernatant with a Pierce LDH cytotoxicity assay kit. All samples were blank corrected. The control was cell culture medium spiked with LDH provided with the kit. Values represent means with standard deviations from three independent experiments. Download FIG S1, PDF file, 0.5 MB. Copyright © 2020 Sobieraj et al. 2020 Sobieraj et al. This content is distributed under the terms of the Creative Commons Attribution 4.0 International license.

### Fusion to ABD extends serum circulation half-life of PGHs.

**FIG 5:**
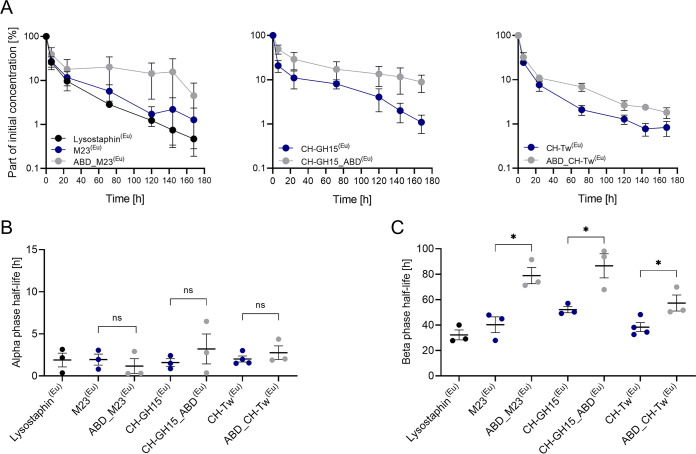
Pharmacokinetic analysis of parental and ABD-fused PGH constructs in mice. (A) Normalized PGH concentrations in the murine plasma over time. Europium-labeled PGHs were injected into mice via the tail vein. PGH concentrations were monitored for 164 h by TRF. (B) Alpha (distribution)-phase half-lives of the tested PGHs. (C) Beta (elimination)-phase half-lives of the tested PGHs. Half-lives of tested proteins were calculated according to a two-compartment model using PK-solver software (61). Asterisks indicate statistical significance (*, *P* < 0.05; ns, nonsignificant, *P* > 0.05).

We next investigated whether ABD fusion would extend the serum circulation half-life of our PGH constructs in mice. For this purpose, parental and ABD-fused enzymes were labeled with europium (Eu) for detection by time-resolved fluorescence (TRF). Possible side effects of Eu labeling on lytic activity and binding to mouse serum albumin were determined for CH-GH15 and CH-GH15_ABD (Fig. S2). We found that neither binding to MSA (Fig. S2A) nor activity of the PGHs (Fig. S2B) was significantly affected by the labeling procedure. Then, highly purified and endotoxin-free (<1 endotoxin unit [EU]/ml) Eu-labeled PGHs were injected into mice via the tail vein, and PGH concentrations in the bloodstream were monitored by TRF. The change in normalized mean PGH serum concentrations over time is presented in Fig. 5A. The biphasic shape of the curves suggests that PGHs followed a two-step process of elimination from the bloodstream, which can be described by a two-compartmental pharmacokinetic model. The model assumes that a metabolite is removed from the blood in the following two phases: (i) distribution, also referred to as the alpha phase, which is characterized by an initial rapid decrease in concentration of the PGH; and (ii) elimination, also referred to as the beta phase (60). The distribution phase lasted about 24 h for all tested PGHs, consistent with previous observations (41). At 72 h postinjection, the concentration of all tested parental enzymes in murine serum had dropped below 8.1% of the initial concentration. At the same time point, ABD-fusion PGHs ABD_M23 and CH-GH15_ABD remained at much higher levels, i.e., 20.1% and 17.3% of the initial concentration, respectively. ABD_CH-Tw was eliminated from murine plasma at a higher rate than the other ABD fusion proteins; however, its concentration at 72 h postinjection was still more than 3.4-fold higher than that of the corresponding parental PGH CH-Tw. The levels of all ABD-fused PGHs in the murine serum remained higher than those of their respective parental constructs until the end of the experiment. Alpha-phase half-lives ranged from 1.17 h to 3.2 h, and there were no significant differences between the tested PGHs (Fig. 5B). Lysostaphin was included as a reference in this experiment, and its alpha-phase half-life was found to be 1.9 h, consistent with previous findings (36, 37). The beta-phase half-life values for all tested ABD-fused PGHs were found to be significantly higher than those of the respective parental enzymes, which ranged between 32 and 52 h (Fig. 5C). The highest half-life values (79 h and 87 h) were observed for ABD_M23 and CH-GH15_ABD, respectively. These values were approximately two times higher than those observed for the respective parental constructs. The beta-phase half-life of ABD_CH-Tw was 1.5-fold longer than that of its parental enzyme. Furthermore, we investigated the biodistribution of PGHs in murine livers and kidneys (Fig. S3). Higher concentrations of the parental PGHs M23 and CH-GH15 than of the respective ABD fusion constructs were detected in livers and kidneys, as reported before for another PGH (41), although the differences were not statistically significant. CH-Tw and ABD_CH-Tw were found in comparable amounts in both organs.

10.1128/mBio.01781-20.2 FIG S2 Influence of conjugated europium label (DTBTA-Eu^3+^) on serum albumin binding and lytic activity of PGHs. (A) Binding of native and Eu-labeled ABD-fused CH-GH15 [CH-GH15_ABD and CH-GH15_ABD^(EU)^] to immobilized mouse serum albumin (MSA), as determined by SPR. Association was measured for 180 s and dissociation for 720 s at a constant flow rate of 10 μl/min. Injections of native (CH-GH15) and Eu-labeled [CH-GH15^(Eu)^] parental enzymes at a concentration of 20 nM served as controls. (B) Analysis of S. aureus lysis by native and Eu-labeled PGHs in TRAs. Enzymes at a concentration of 50 nM were mixed with S. aureus Newman cells, and the reduction in optical density at 600 nm was monitored over time. Error bars represent standard errors of the means of results from four individual experiments. ns, nonsignificant. Download FIG S2, PDF file, 0.6 MB. Copyright © 2020 Sobieraj et al. 2020 Sobieraj et al. This content is distributed under the terms of the Creative Commons Attribution 4.0 International license.

10.1128/mBio.01781-20.3 FIG S3 Biodistribution of europium-labeled PGHs in murine livers and kidneys. Eu-labeled PGHs were injected into the mice via the tail vein (5 mg/kg body weight). After 168 h, mice were sacrificed and livers and kidneys were collected and homogenized. The concentration of PGHs in the organs was determined by TRF. Error bars represent standard errors of the means of results from two [CH-Tw^(Eu)^] or three (all other enzymes) individual experiments. Asterisks indicate statistical significance (**, *P* < 0.01; ns, nonsignificant, *P* > 0.05). Download FIG S3, PDF file, 0.4 MB. Copyright © 2020 Sobieraj et al. 2020 Sobieraj et al. This content is distributed under the terms of the Creative Commons Attribution 4.0 International license.

### ABD-fused PGH features improved *in vivo* efficacy in a murine model.

**FIG 6:**
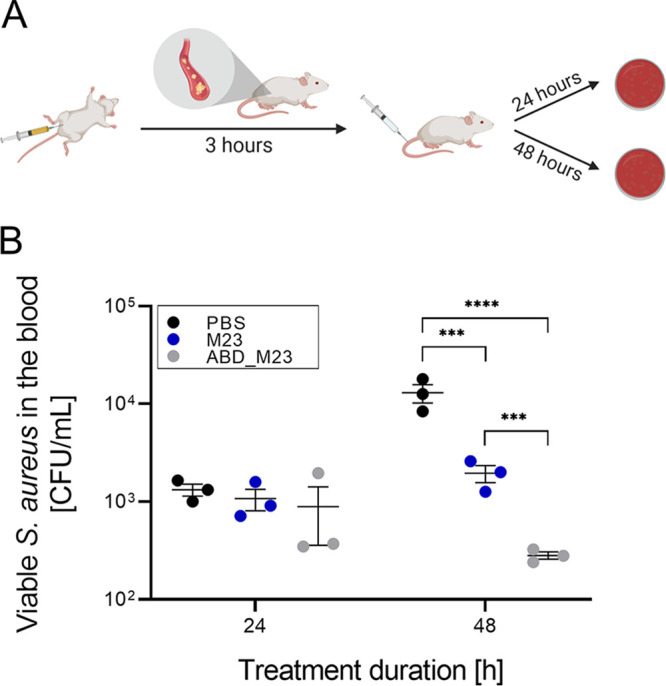
*In vivo* efficacy of M23 and ABD_M23 PGHs in a model of murine bacteremia. (A) Schematic outline of the performed experiment. Mice were intraperitoneally infected with S. aureus Cowan and treated with a single dose (100 μl) of 1 μM M23 or ABD_M23 or PBS as a control, administered intravenously 3 h postinfection. Viable bacterial counts in the blood were monitored at 24 h and 48 h posttreatment. The figure panel was created with BioRender software. (B) Viable S. aureus in the murine bloodstream at 24 h and 48 h post-PGH treatment. Control mice received sterile PBS. Error bars indicate standard errors of the means of results from three biological replicates. The detection limit was 100 CFU/ml. Asterisks indicate statistical significance (***, *P* < 0.001; ****, *P* < 0.0001).

Finally, we investigated whether fusion of an ABD to a PGH would result in enhanced therapeutic efficacy. To this end, the enzymes M23 and ABD_M23 were evaluated in a mouse model of S. aureus*-*induced bacteremia. This pair of enzymes was selected because ABD_M23 had demonstrated high staphylolytic activity *in vitro* and *ex vivo* and had shown the highest improvement in half-life compared to its parental enzyme. Here, mice were infected with S. aureus Cowan by injection into the peritoneum. Three hours postinfection, mice were treated with one intravenous dose of parental or ABD-fused PGH or PBS (depicted in Fig. 6A). The bacterial load in the bloodstream of infected mice was monitored at 24 and 48 h posttreatment (Fig. 6B). After 24 h, the bacterial concentration in the blood reached approximately 10^3^ CFU/ml, with no significant differences between the treatment groups. After 48 h, the bacterial loads in the parental PGH and ABD-fusion treatment groups were strongly reduced compared to the control group (*P* = 0.0008 and *P* < 0.0001, respectively). Of note, bacterial counts in the mice treated with the ABD-fusion construct were significantly lower (*P* = 0.0008) than in those treated with an equimolar amount of the parental PGH construct.

## DISCUSSION

Half-life extension by ABD fusion has been successfully pursued for a number of protein therapeutics (49). It also constitutes a promising strategy for improvement of pharmacokinetic properties of PGHs, as demonstrated here and in previous proof-of-principle studies (37, 41). In our current study, the long-circulating PGH ABD_M23 construct proved superior to its parental M23 enzyme in killing S. aureus in the bloodstream of bacteremic mice, despite its reduced activity *in vitro.* It seems reasonable to assume that this can be attributed to the longer terminal half-life of ABD_M23 and, therefore, to a higher effective concentration of the engineered PGH in the bloodstream during the elimination phase. However, the possibility cannot be excluded that other factors besides half-life extension were responsible for or contributed to the observed outcome of the mouse study. These include (i) a possibly higher stability of ABD_M23 in the murine system compared to parental M23, which would lead to a higher proportion of active ABD_M23 after 48 h; (ii) a possibly better dissemination within the bloodstream of ABD_M23 when bound to albumin; (iii) a possibly closer localization of ABD_M23 to S. aureus due to binding of the bacteria to albumin; and (iv) a possible increase in ABD_M23 activity due to synergistic interaction with other host factors present in murine blood, as was previously described for the PGH CF-301 in human and rabbit blood (58). Further studies are required to investigate the possible roles of these factors in the enhanced efficacy of the ABD-tagged enzyme in mice. Of note, at 24 h posttreatment, which corresponds to the approximate end of the distribution phase, we did not observe significant differences in the bacterial loads between the two treatment groups. This may be accredited to the fact that distribution half-lives were not influenced by ABD recruitment and that the *ex vivo* whole-blood killing efficacies of ABD_M23 and M23 were highly similar over the 24-h time span (Fig. 4). Given the rapid killing of S. aureus by both enzymes *ex vivo*, it seems surprising that the bacterial concentrations in enzyme-treated mice did not differ significantly from those in control mice at 24 h (Fig. 6B). This could possibly be explained by the lower enzyme concentration in the murine bloodstream (approximately 50 nM, assuming an average murine blood volume of 2 ml) than in the *ex vivo* experiment (100 nM); the fact that elimination of the enzymes started immediately upon injection into the mice, whereas it remained constant *ex vivo*; and our finding that the antibacterial activity of M23 was significantly reduced in mouse serum compared to human serum (data not shown). The latter is in agreement with the reportedly lower activity of CF-301 in mouse blood compared to human blood (58).

To date, half-life extension of PGHs had been attempted via multiple approaches, including dimerization (38, 45), PEGylation (36), and ABD fusion (37, 41). Previous studies were able to show improvements in the PGH pharmacokinetic (36–38, 41, 45) or pharmacodynamic (37) properties but not the therapeutic implications of a prolonged half-life. The presumable reason for that was either a complete loss (44) or at least a significant decrease in the activity of the modified PGHs (36, 37, 41, 45), in which a prolonged half-life would likely have not been able to compensate for lost activity. By altering the intramolecular position of the ABD in the context of the PGH (i.e., at an N- or C-terminal position), as well as by the introduction of a specific endolysin-originating (52) connecting linker sequence, we were able to provide constructs that retained significant staphylolytic activity in the presence of HSA. Notably, we found that all of the enzymes responded differently to modifications and that each featured a unique and individual optimized architecture.

Nevertheless, a reduced activity for all ABD fusions compared to the parental enzymes was observed in *in vitro* assays (particularly in TRAs), as shown before (37, 41). Moreover, in the presence of albumin, the activity further decreased for some but not all ABD-fused PGH variants, whereas it was boosted for the respective parental constructs, as reported previously for other enzymes (41, 58). This increase may be explained by the ability of excess HSA to prevent unspecific binding of the parental PGHs to the microplate surface, thereby maintaining the PGH concentration in solution. With respect to the ABD-fused variants, bulky complexes with HSA might sterically hinder binding to or enzymatic cleavage of the bacterial peptidoglycan by the PGH or both, representing two processes that are essential for lysis of the target bacteria. All ABD-fusion constructs created here showed nano- to picomolar affinity to HSA, which was less than the femtomolar binding of the ABD alone (47). Additionally, there were discrepancies in the strength of HSA binding between the different ABD-fusion PGH constructs. Similarly to the effects on lytic activity, lower and differing affinities could be a result of steric hindrance intrinsic to these fusion proteins. This could possibly be controlled by the linker length and/or position of the ABD within the PGH in a protein-specific manner. We found that the ABD-PGH with the highest affinity toward HSA (i.e., CH-GH15_ABD) showed the longest serum circulation half-life *in vivo* but also showed the lowest activity in the presence of albumin. In contrast, the ABD-fused PGH that showed the highest activity (i.e., ABD_CH-Tw) bound HSA with the lowest affinity and displayed the shortest serum circulation half-life. This suggests that weaker binding to albumin in ABD-fusion constructs might have a positive effect on their activity due to reduced steric hindrance but at a cost of a shorter *in vivo* half-life. For the best *in vivo* efficacy, these aspects should be considered when designing long-circulating ABD-fusion PGHs in the future.

As mentioned above, the ABD fusion strategy to extend PGH serum circulation half-life (37, 41) appears superior to other approaches, such as PEGylation (36, 44) and dimerization (38, 45). Both of the latter strategies also increase the protein’s molecular radius, thereby reducing the kidney excretion rate and, consequently, the elimination rate. However, chemical conjugation of polyethylene glycol (PEG) chains also affected activity of lysostaphin (36) and Cpl-1 (44) tremendously. Therefore, despite the prolonged half-life and reduced immunogenicity, PEGylated PGHs have not been tested for their *in vivo* efficacy. Moreover, while the process of construction, expression, and purification of ABD-fused proteins is very straightforward, PEGylation is based on a difficult-to-control chemical modification that yields heterogeneous products and requires additional purification steps. Resch et al. reported that dimerization of Cpl-1 lead to an increased activity and a reduced clearance rate of this endolysin (38). In contrast, dimerized lysostaphin showed a substantially reduced activity and, despite improved pharmacokinetic properties, can be expected to be inferior to the parental enzyme in tests performed *in vivo* (45). Thus, while dimerization may be a viable half-life extension strategy, it should be applied only for PGHs that have a natural tendency to form multimers and that therefore might not suffer a drastic loss in activity. In contrast, ABD fusion has led to 1.5-fold to 5-fold increases in terminal half-lives of PGHs, including two-domain enzymes of bacterial and phage origins {i.e., lysostaphin (37) and CSL[Eu] (41), respectively} as well as chimeric enzymes featuring two domains (i.e., M23, CH-GH15) or three domains (i.e., CH-Tw) as in this study. Thus, it is reasonable to assume that, unlike dimerization, ABD fusion could be successfully applied to various PGHs, regardless of their origin, architecture, and biochemical properties.

It is interesting that, in this and our previous work (41), the terminal serum circulation half-lives of parental PGHs ranged between 23 and 52 h and were thus unexpectedly long. Earlier studies (36–40, 45) reported much shorter half-lives of the parental PGHs. We have observed that PGHs follow a two-step process of elimination from the bloodstream. Using a two-compartment model analysis, we determined that the initial 24 h postinjection corresponded to the distribution phase, in which the enzyme concentration in the bloodstream drops rapidly as a result of its dissemination throughout the body (60). In the earlier studies, PGH concentrations in the bloodstream had been measured for only 24 h postinjection; hence, the reported half-lives might have corresponded only to the enzymes’ distribution rather than to their elimination. Therefore, it seems plausible that the actual terminal serum circulation half-lives of PGHs had previously been underestimated.

In the past, research on peptidoglycan-degrading enzymes had been focused mainly on characterizing and engineering arbitrarily selected PGH candidates using artificial conditions that may not reflect realistic scenarios (discussed in references 50 and 61). Here, we applied a previously developed screening protocol (50) to first identify PGHs featuring high activity in human serum, followed by engineering for extended half-lives. This approach allowed for qualitative but not quantitative comparison of enzymes, due to the variety of strains and vectors present in the library, which could possibly cause differences in the expression levels and solubility of the tested proteins. To correct for this potential bias, we iterated multiple activity assays to validate the results for the top candidate enzymes. This comprehensive selection process resulted in three PGHs highly active against multiple S. aureus strains in complex environments such as human serum and whole blood. We believe that systematic and rigorous selection of candidate enzybiotics was paramount for the success of this study. Notably, the enzymes M23 and ABD_M23 chosen for evaluating *in vivo* efficacy were applied at extremely low doses in the murine bacteremia model, i.e., at concentrations several magnitudes lower than those used in other studies on S. aureus bacteremia (28, 34). Despite this, both PGHs significantly reduced S. aureus viable counts in the bloodstream of bacteremic mice over a 48-h period.

In conclusion, our study demonstrated that fusion of an ABD to a PGH not only improves its pharmacokinetic properties but also results in enhanced efficacy in treating S. aureus bacteremia *in vivo*. We believe that our results constitute an important advancement in the development of PGH-based antibacterial agents for systemic applications.

## MATERIALS AND METHODS

### Bacterial strains and growth conditions.

Bacterial strains used in this study are listed in Table S3 in the supplemental material. S. aureus strains were grown at 37°C in tryptic soy broth (TSB). Escherichia coli strains were cultured at 37°C in LB or LB-PE for protein expression (62). E. coli ClearColi BL21(DE3) (Lucigen, Middleton, WI, USA) was cultured according to the manufacturer’s recommendations. LB media were supplemented with appropriate antibiotics, depending on the plasmid used (Table S1). E. coli strains harboring pET302, pET21a, and pQE30 vectors were grown in the presence of ampicillin (100 μg/ml), and strains carrying a pET9a vector were cultured in the presence of kanamycin (50 μg/ml).

10.1128/mBio.01781-20.6 TABLE S3 Bacterial strains used in this study. Download Table S3, PDF file, 0.5 MB. Copyright © 2020 Sobieraj et al. 2020 Sobieraj et al. This content is distributed under the terms of the Creative Commons Attribution 4.0 International license.

### Protein expression and purification.

Proteins were expressed according to a previously published protocol (41). Bacteria were lysed using a Stansted Fluid Power pressure cell homogenizer (100 MPa). Proteins harboring His tags were purified by nickel affinity chromatography as described elsewhere (29). Proteins without His tags were purified by cation exchange chromatography (CIEX) using a 5-ml HiTrap SP-FF column on a fast protein liquid chromatography (FPLC) device (Äkta purifier; GE Healthcare Life Sciences). Proteins used for the *in vivo* experiments were expressed in E. coli ClearColi BL21(DE3) as described elsewhere (41). In this case, protein purification was performed in an endotoxin-free environment. The purification columns and chromatography system were decontaminated using 1 M NaOH prior to sample loading. Additional purification by size exclusion chromatography (SEC) was performed following the CIEX step (Superdex 200 Increase 10/300 GL; GE Healthcare) using SEC running buffer (50 mM Na_2_HPO_4_, 500 mM NaCl, pH 7.4). Proteins were tested for endotoxin content using an EndoZyme kit (Hyglos, Regensburg, Germany) according to the manufacturer’s instructions. Protein identity and purity were confirmed by SDS-PAGE, followed by Coomassie staining (InstantBlue; Sigma).

### Endolysin functional assays.

Time-kill assays (TKAs) were performed as previously described (41). PBS or human serum (Sigma) (human male AB plasma, U.S. origin, sterile filtered) was spiked with early-log-phase S. aureus and mixed with lytic enzymes. Samples were incubated at 37°C. PBS was used as a negative control. Viable bacterial counts were monitored at different time points by serial dilution plating. Turbidity reduction assays (TRAs) were performed essentially as described before (29, 41). Briefly, frozen but viable S. aureus Newman substrate cells were resuspended in PBS, in PBS supplemented with 5 μM HSA, or in 100% human serum and were mixed with equimolar concentrations of PGHs. Reductions in turbidity were monitored over time. Specific enzymatic activity was calculated as described elsewhere (21). MIC assays were conducted according to the standard protocol (63) with alterations. S. aureus Newman was grown until an optical density (OD) of 0.5 was reached. The culture was diluted in TSB with human serum (mixture of 20% 5× TSB and 80% human serum) to 1 × 10^6^ CFU/ml. Two-fold dilution of the lytic enzymes was performed in TSB with human serum in a 96-well microplate. Diluted enzymes were mixed at a 1:1 ratio with the bacterial suspension. Plates were incubated for 18 h at 37°C without shaking. Stability assays (SAs) were performed in human serum at 37°C. Lytic enzymes were diluted in human serum and incubated at 37°C for 4 h. Activity of the enzymes before and after incubation was determined by TRA. Values representing changes in activity levels measured as optical density changes (ΔOD_600_) per minute were normalized to the control, and enzyme stability was expressed as a percentage of the activity remaining after incubation.

### Screening and selection of PGHs with high activity in human serum.

Parental and engineered PGHs from our laboratory collection (*n* = 317) were screened for activity against S. aureus in human serum. Protein expression experiments were performed in a 96-well format as described elsewhere (50). Chloroform-lysed cells containing the PGH of interest were exposed to human serum spiked with S. aureus Newman. Aliquots from each well were spotted on TSB agar and incubated overnight at 37°C. PGH selection and elimination were iterative processes. In step 1, 317 constructs were challenged with human serum spiked with 10^5^ CFU/ml bacteria for 2 h. In step 2, 150 top-performing PGHs were challenged with 10^7^ CFU/ml bacteria for 2 h. In step 3, the 77 best-performing PGHs were challenged with 10^7^ CFU/ml bacteria for 30 min. In all steps, activity of the PGH constructs was assessed by comparisons of the S. aureus colony densities within the individual spots with that of the controls. E. coli strains harboring a lysostaphin gene and an empty pET21a vector served as positive and negative controls, respectively. Selected enzymes were expressed and purified and further analyzed by TKA, TRA, MIC assay, and SA. All assays were performed in human serum using S. aureus Newman as a target bacterium. In TKA, enzymes were tested at 20 nM concentration against 10^6^ CFU/ml of S. aureus. Log reduction in viable bacterial counts was determined after 10, 30, and 60 min. Results of each assay were normalized to the highest scorer, and scores between 0 and 10 were assigned to each protein. The overall score was calculated based on a weighted sum of scores from TKA, TRA, MIC, and SA results, which were assigned according to the importance of each assay as follows: 4×TKA + 2×MIC + 2×TRA + 2×SA. Enzymes were grouped into the following categories based on their cleavage specificity: (i) Gly-Gly endopeptidases, (ii) Gly–d-Ala endopeptidases, and (iii) double- and triple-acting enzymes. The highest-scoring enzyme from each category was selected for further experiments. Selected PGHs were tested against other MSSA (Cowan and SA113) and MRSA (USA300 JE2, ZH123, and ZH313) strains (Table S3) in a TKA at a 100 nM concentration and a 10^6^ CFU/ml starting bacterial load in human serum.

### Design and construction of albumin-binding PGH variants.

Selected PGHs were fused to an affinity-matured variant of an albumin-binding domain (ABD), ABD035 (47). The ABD was linked with the PGHs via a flexible 33-amino-acid (33-aa) linker (L33) from the phage endolysin Ply2638A (aa 360 to aa 392 region) (52). The first generation of PGH-ABD constructs featured C-terminal ABD fusions, since an N-terminal fusion in LysK had previously proven inferior (41). However, these constructs displayed rather poor activity compared to the parental enzymes. Therefore, a second generation of ABD fusion PGHs featuring N-terminal ABDs was constructed. The ABD was followed by the L33 linker or a duplicated 66-amino-acid version of it (L66). Construction of the ABD-fusion variants was performed according to standard molecular cloning protocols (64).

### Characterization of the albumin-binding PGH variants.

Levels of lytic activity of the created ABD fusion constructs and the respective parental enzymes were compared in TRAs and TKAs using S. aureus Newman as a target organism, essentially as described previously (41). TRAs were conducted in PBS and in PBS supplemented with 5 μM HSA. TKAs were performed in human serum with 10^5^ CFU/ml starting bacterial load and 100 nM enzyme concentration. Affinity of the most active ABD-fused PGHs to immobilized HSA was tested by surface plasmon resonance (SPR) in a Biacore X system (GE Healthcare Life Sciences). HSA (70 μl, 2 μg/ml) was immobilized on a CMD500L chip (XanTec) by amino coupling according to the recommendations of the manufacturer at a flow rate of 5 μl/min in flow cell 2 (Fc2). Sodium acetate at pH 4.2 was used as an immobilization buffer and 1-ethyl-3-(3-dimethylaminopropyl)-carbodiimide (EDC) and N-hydroxysuccinimide (NHS) at a 1:1 ratio for activation of the surface. No protein was immobilized in flow cell 1 (Fc1), which served as a reference. Dilutions (20 nM, 10 nM, 5 nM, and 1 nM) of the ABD-fused variants were prepared in SPR running buffer (10 mM HEPES, 150 mM NaCl, 3.4 mM EDTA, 0.005% Tween 20, pH 7.4), and a 30-μl volume of each was injected at a flow rate of 10 μl/min. The surface was regenerated using 5 μl of 10 mM HCl between the samples. Injections of the respective parental enzymes at 20 nM concentration served as controls. Corrected (Fc2-Fc1) sensorgrams were analyzed using BiaEvaluation software (GE Healthcare Sciences) and the mass-transfer model (65). Additionally, binding of the ABD fusion constructs to HSA in solution was examined through an electrophoretic mobility shift assay (EMSA). Parental and ABD-fused PGH constructs in a mixture containing PBS were mixed with HSA at 1:1.2 molar ratios. The mixtures were incubated for 10 min at room temperature. Native PAGE was performed using Any kD TGX precast gels (Bio-Rad) in Tris-glycine buffer (25 mM Tris, 192 mM glycine, pH 8.3) for 30 min at 150 V. The high isoelectric points (>9.5) of the PGHs used required reversed current in the system since the proteins in the native conformation migrated toward the cathode.

### Determination of cytotoxicity of PGH constructs.

MG-63 (ATCC CRL-1427) osteosarcoma cells (MG-63 cells) were maintained at ≤ 90% confluence in minimum essential medium (MEM; Gibco)–Earl’s salts–l-glutamine–10% fetal bovine serum (FBS) and incubated at 37°C and 5% CO_2_ (66). Cytotoxicity assays were performed using a Pierce lactate dehydrogenase (LDH) cytotoxicity assay kit (Thermo Fisher Scientific, Waltham, USA) according to the manufacturer’s instructions with a PGH concentration of 2 μM and an exposure time of 22 h. Cell culture medium spiked with LDH provided with the kit was used as a positive control.

### *Ex vivo* activity testing of PGH constructs in whole human blood.

Blood samples from healthy volunteers (20 to 40 years of age, male and female) were collected at the Department of Infectious Diseases and Hospital Epidemiology at the University Hospital Zurich. All healthy volunteers were included after providing signed informed consent, and experiments were performed in accordance with the Declaration of Helsinki and with permission from the Cantonal Ethics Committee Zurich (Kantonale Ethikkommission Zürich), Zurich, Switzerland. Human whole-blood killing was performed by modifying a previously described procedure (67). In short, blood was aliquoted into low-protein binding tubes (Protein LoBind; Eppendorf) and spiked with early-log-phase S. aureus Newman, Cowan, or USA300 JE2 at a concentration of 10^4^ CFU/ml. Parental and ABD-fused PGH constructs were added at a concentration of 100 nM. Samples were incubated at 37°C with constant mixing. Viable counts of bacteria were determined by plating serial dilutions of blood in MilliQ water at defined time points.

### PGH labeling with fluorescent europium chelate DTBTA-Eu^3+^.

Protein labeling with the fluorescent lanthanide europium was performed essentially as described before (68). Sodium [4′-(4′-amino-4-biphenylyl)-2,2′:6′,2′'-terpyridine-6,6′'-diylbis(methyliminodiacetato)]europate(III) (ATBTA-Eu^3+^) (TCI Chemicals) was transformed into [21′′′-{4′-{[(4,6-dichloro-1,3,5-triazin-2-yl)amino]biphenyl-4-yl]-2,2′:6′,2′′-terpyridine-6,6′′-iyl}bis-(methylenenitrilo)}tetrakis(acetato)}europium(III) (DTBTA-Eu^3+^), as instructed by the manufacturer. DTBTA-Eu^3+^ was mixed with the purified proteins at a molar ratio of 3:1. The protein–DTBTA-Eu^3+^ mixture containing carbonate buffer (pH 9.2) supplemented with 300 mM NaCl and 10% glycerol was incubated for 2.5 h with constant mixing at room temperature. Unbound DTBTA-Eu^3+^ and cross-linked protein species were removed by SEC (HiLoad Superdex 200 16/600 column; GE Healthcare). The concentration of labeled proteins was determined with a Pierce bicinchoninic acid (BCA) protein assay kit (Thermo Fisher Scientific) according to the manufacturer’s instructions. Selected labeled proteins were assayed for their activity (as determined by TRA) and binding to mouse serum albumin (MSA) (measured by SPR) to test if the labeling had altered the proteins’ properties.

### Pharmacokinetics and biodistribution study.

All *in vivo* experiments were performed in accordance with the guidelines of the institutional animal care and use committee of the University of Zurich. All animal experiments conducted in this study were approved by the Cantonal Veterinary Office Zurich, Zurich, Switzerland (protocols ZH251/14 and ZH050/18). C57BL/6 mice (female, 8 to 10 weeks of age) were injected with 100 μl of the europium-labeled PGH preparations in a mixture containing Dulbecco's phosphate-buffered saline (DPBS) at a concentration of 1 mg/kg of body weight (*n* = 2 for CH-Tw) or 5 mg/kg (*n* = 2 for CH-Tw and *n* = 3 for other PGHs) into the tail vain. Blood was drawn from the tail vein after 0.25 h, 6 h, 24 h, 72 h, 120 h, 144 h, and 168 h postinjection. At 168 h postinjection, the mice were sacrificed. Kidneys and livers were collected, diluted 1:1 in PBS, and homogenized. Concentrations of europium-labeled PGHs in the samples were determined by time-resolved fluorescence (TRF) (Tecan Infinite M1000 plate reader) (excitation wavelength, 348 nm; lag time, 275 μs; emission wavelength, 619 nm; integration time, 500 μs). Half-lives of the tested proteins were calculated for each replicate from the absolute values of the serum concentrations according to a two-compartment model using PK-solver software (69).

### *In vivo* efficacy evaluation.

Female C57BL/6 mice (8 to 10 weeks of age) were infected with 0.8 × 10^8^ to 1.5 × 10^8^ CFU of S. aureus Cowan by intraperitoneal injection. At 3 h postinfection, mice were treated with 100 μl of M23LST(L)_SH3b2638A (*n* = 3) or ABD(L33)_M23LST(L)_SH3b2638A (*n* = 3) at a concentration of 1 μM administered via the tail vein. Control mice received 100 μl of sterile DPBS (*n* = 3). Blood was drawn from the tail vein 24 h and 48 h posttreatment. Collected blood aliquots were plated on blood agar to evaluate the load of S. aureus in the bloodstream. Mice were sacrificed 48 h posttreatment.

### Statistical analysis.

Statistical analyses were performed in GraphPad Prism 8.2.0 (GraphPad Software, San Diego, CA, USA). TKA analysis was performed on log-transformed CFU counts using an ordinary one-way analysis of variance (ANOVA) followed by Dunnett's multiple-comparison test. A one-way ANOVA followed by Tukey's multiple-comparison test was performed to analyze the specific activities of different ABD-fusion constructs and parental enzymes in PBS and PBS-HSA, as determined by TRA. Activities of the europium-labeled and native proteins, half-lives, and organ concentrations of the parental and ABD-fused PGHs were compared with an unpaired Student's *t* test. One-way ANOVA followed by Tukey's multiple-comparison test was performed on log-transformed CFU counts in murine blood. *P* values of less than 0.05 were considered statistically significant.

### Data availability.

We declare that the data supporting the findings of this study are available within the article and its supplemental material files or from the corresponding author on request.
