# Supplementary material for: Engineering of Long-Circulating Peptidoglycan Hydrolases Enables Efficient Treatment of Systemic Staphylococcus aureus Infection
Source: mBio. 2020 Sep 22;11(5):e01781-20. doi: 10.1128/mBio.01781-20 (PMC7512550; doi:10.1128/mBio.01781-20)
Supplement: TABLE S1 [file mBio.01781-20-st001.pdf]

Table S1. List of peptidoglycan hydrolases (PGHs) used in the study. PGHs 1-25 were part of the laboratory collection. ABD-fused PGHs were constructed in this study.

ID / Acronym	PGH construct name	Vector	Strains
PGH-1 / LST	Lysostaphin	pET9a	*E. coli* BL21Gold (DE3)<br>ClearColi® BL21 (DE3)
PGH-2 / M23	M23LST(L)_SH3b2638A	pET9a	*E. coli* BL21Gold (DE3)<br>ClearColi® BL21 (DE3)
PGH-3	M23LST_SH3b2638A	pET302	*E. coli* BL21Gold (DE3)
PGH-4	(M23LST)$_2$_SH3b2638A	pET302	*E. coli* BL21Gold (DE3)
PGH-5	(M23LST)$_2$_SH3bAle1	pET302	*E. coli* BL21Gold (DE3)
PGH-6	H_TEV_(M23LST)$_2$_SH3bLST	pQE30	*E. coli* Sure
PGH-7	H_(LST)$_2$	pQE30	*E. coli* Sure
PGH-8	H_M23LST_SH3bLST_ M23LST	pQE30	*E. coli* Sure
PGH-9	CHAPTw_SH3b2638A	pET302	*E. coli* BL21Gold (DE3)
PGH-10	H_CHAP-1_CBD-1	pET302	*E. coli* BL21Gold (DE3)
PGH-11 / CH-GH15	CHAPGH15_SH3bALE1	pET302	*E. coli* BL21Gold (DE3)<br>ClearColi® BL21 (DE3)
PGH-12	CHAPK_SH3bLST_H	pET21a	*E. coli* BL21Gold (DE3)
PGH-13	CHAP187_SH3bK_H	pET21a	*E. coli* BL21Gold (DE3)
PGH-14	(CHAPGH15)$_2$_SH3b2638A	pET302	*E. coli* BL21Gold (DE3)
PGH-15	(CHAPGH15)$_2$_SH3bALE1	pET302	*E. coli* BL21Gold (DE3)
PGH-16	H_Xa_(CHAPTw)2_SH3b2638A	pQE30	*E. coli* Sure
PGH-17	H_Xa_(CHAPTw)2_SH3bLST	pQE30	*E. coli* Sure
PGH-18 / CH-Tw	CHAPTw_M23LST(L)_SH3b2638A	pET9a<br>pET302	*E. coli* BL21Gold (DE3)<br>ClearColi® BL21 (DE3)
PGH-19	M23LST_Ami2638A_SH3b2638A	pET302	*E. coli* BL21Gold (DE3)
PGH-20	CHAPK_AmiK_SH3bLST_H	pET21a	*E. coli* BL21Gold (DE3)
PGH-21	CHAPH5_LST_H	pET21a	*E. coli* BL21Gold (DE3)
PGH-22	LST_CHAPK_AmiK_H	pET21a	*E. coli* BL21Gold (DE3)
PGH-23	CHAPH5_AmiH5_LST_H	pET21a	*E. coli* BL21Gold (DE3)
PGH-24	LST_CHAPH5_AmiH5(L)_H	pET21a	*E. coli* BL21Gold (DE3)
PGH-25	CHAP11_Ami11_LST_H	pET21a	*E. coli* BL21Gold (DE3)
M23_(L33)ABD	M23LST(L)_SH3b2638A_(L33)ABD035	pET302	*E. coli* BL21Gold (DE3)
ABD(L33)_M23	ABD035(L33)_M23LST(L)_SH3b2638A	pET302	*E. coli* BL21Gold (DE3)<br>ClearColi® BL21 (DE3)
ABD(L66)_M23	ABD035(L66)_M23LST(L)_SH3b2638A	pET302	*E. coli* BL21Gold (DE3)
CH-GH15_(L33)ABD	CHAPGH15_SH3bALE1_(L)_ABD035	pET302	*E. coli* BL21Gold (DE3)<br>ClearColi® BL21 (DE3)
ABD(L33)_CH-GH15	ABD035(L33)_CHAPGH15_SH3bALE1	pET302	*E. coli* BL21Gold (DE3)
ABD(L66)_CH-GH15	ABD035(L66)_CHAPGH15_SH3bALE1	pET302	*E. coli* BL21Gold (DE3)
CH-Tw_(L33)ABD	CHAPTw_M23LST(L)_SH3b2638A_(L)ABD035	pET302	*E. coli* BL21Gold (DE3)
ABD(L33)_CH-Tw	ABD035(L33)_CHAPTw_M23LST(L)_SH3b2638A	pET302	*E. coli* BL21Gold (DE3)
ABD(L66)_CH-Tw	ABD035(L33)_CHAPTw_M23LST(L)_SH3b2638A	pET302	*E. coli* BL21Gold (DE3)<br>ClearColi® BL21 (DE3)
