# Supplementary material for: Engineering of Long-Circulating Peptidoglycan Hydrolases Enables Efficient Treatment of Systemic Staphylococcus aureus Infection
Source: mBio. 2020 Sep 22;11(5):e01781-20. doi: 10.1128/mBio.01781-20 (PMC7512550; doi:10.1128/mBio.01781-20)
Supplement: TABLE S2 [file mBio.01781-20-st002.pdf]

Table S2. Characterization of PGHs identified in the screening by time-kill (TKA), turbidity reduction (TRA), minimum inhibitory concentration (MIC) and stability (SA) assays. Results of each assay were normalized to the highest scorer. The final score was calculated based on a weighted sum: 4×TKA + 2×MIC + 2×TRA + 2×SA. Enzymes were grouped into three categories based on their cleavage specificity: (i) Gly - Gly endopeptidases, (ii) Gly - D-Ala endopeptidases, (iii) double- and triple-acting enzymes. The highest-scoring enzyme from each category (shown in bold) was selected for further experiments. ND – not determined.

			**TKA**					**TRA**		**MIC**		**SA**		**Final score**
			log reduction				rating	specific activity [$\Delta ODmin^{-1}\mu M^{-1}$]	rating	MIC [nM]	rating	remaining activity [%]	rating	4×TKA + 2×MIC + 2×TRA + 2×SA
			10 min	30 min	60 min	average								
Gly - Gly endopeptidases	PGH 1	Lysostaphin	5.86	5.87	5.99	5.91	10.0	1.10	3.6	1.5625	10.0	44.22	4.6	76.4
	PGH 2	**M23LST(L)_SH3b2638**	5.86	5.87	5.99	5.91	10.0	1.35	4.4	3.125	8.9	57.37	6.0	78.6
	PGH 3	M23LST_SH3b2638	4.04	4.87	5.46	4.79	8.1	0.92	3.0	15.625	6.4	53.20	5.5	62.4
	PGH 4	(M23LST)2_SH3b2638	1.69	3.87	4.35	3.30	5.6	0.97	3.1	31.25	5.4	96.20	10.0	65.8
	PGH 5	(M23LST)2_SH3bALE1	3.73	5.35	5.99	5.02	8.5	1.94	6.3	15.625	6.4	55.81	5.8	64.8
	PGH 6	H_TEV_(M23LST)2_SH3bLST	3.04	3.31	4.42	3.59	6.1	1.06	3.5	7.8125	7.5	80.99	8.4	63.1
	PGH 7	H_(LST)2	0.56	2.19	3.76	2.17	3.7	0.14	0.5	15.625	6.4	72.07	7.5	43.5
	PGH 8	H_M23LST_SH3bLST_ M23LST	3.19	4.01	4.46	3.89	6.6	1.15	3.7	62.5	4.3	56.83	5.9	54.2
Gly - D-Ala endopeptidases	PGH 9	CHAPTw_SH3b2638	3.77	4.23	4.46	4.16	7.0	0.87	2.8	15.625	6.4	6.03	0.6	47.9
	PGH 10	CHAP-1_CBD-1	3.96	5.35	5.46	4.92	8.3	0.58	1.9	250	2.1	49.35	5.1	51.7
	PGH 11	**CHAPGH15_SH3bALE1**	3.55	5.87	5.99	5.14	8.7	3.07	10.0	3.125	8.9	78.46	8.2	89.0
	PGH 12	CHAPK_SH3bLST_H	2.56	3.27	3.45	3.09	5.2	0.46	1.5	7.8125	7.5	71.98	7.5	53.9
	PGH 13	CHAP187_SH3bK_H	4.34	5.87	5.99	5.40	9.1	2.32	7.6	62.5	4.3	2.17	0.2	60.7
	PGH 14	(CHAPGH15)2_SH3b2638	-0.01	0.24	1.43	0.55	0.9	0.19	0.6	>1000	0.0	51.30	5.3	15.7
	PGH 15	(CHAPGH15)2_SH3bALE1	2.19	3.57	3.46	3.07	5.2	0.14	0.5	31.25	5.4	20.36	2.1	36.7
	PGH 16	H_Xa_(CHAPTw)2_SH3b2638	0.04	0.12	0.02	0.06	0.1	0.03	0.1	>1000	0.0	ND	0.0	0.6
	PGH 17	H_Xa_(CHAPTw)2_SH3bLST	0.10	0.15	0.26	0.17	0.3	0.07	0.2	>1000	0.0	ND	0.0	1.6
double and triple acting	PGH 18	**CHAPTw_M23LST(L)_SH3b2638**	3.64	5.35	5.99	4.99	8.5	1.18	3.9	15.625	6.4	38.19	4.0	62.3
	PGH 19	M23LST_Ami2638_SH3b2638	1.42	3.05	4.10	2.86	4.8	1.44	4.7	500	1.1	43.87	4.6	40.0
	PGH 20	CHAPK_AmiK_SH3bLST_H	4.30	5.35	5.16	4.94	8.4	0.23	0.8	15.625	6.4	18.06	1.9	51.6
	PGH 21	CHAPH5_LST_H	2.27	3.27	4.46	3.33	5.6	0.26	0.8	15.625	6.4	7.49	0.8	38.7
	PGH 22	LST_CHAPK_AmiK_H	1.51	2.78	4.35	2.88	4.9	0.29	1.0	125	3.2	67.62	7.0	41.9
	PGH 23	CHAPH5_AmiH5_LST_H	0.10	1.93	4.21	2.08	3.5	0.36	1.2	62.5	4.3	68.44	7.1	39.2
	PGH 24	LST_CHAPH5_AmiH5(L)_H	0.03	0.54	2.28	0.95	1.6	0.29	0.9	250	2.1	79.56	8.3	29.2
	PGH 25	CHAP11_Ami11_LST_H	1.27	3.54	5.46	3.42	5.8	0.25	0.8	62.5	4.3	57.55	6.0	45.4
