# Supplementary material for: Engineering of Long-Circulating Peptidoglycan Hydrolases Enables Efficient Treatment of Systemic Staphylococcus aureus Infection
Source: mBio. 2020 Sep 22;11(5):e01781-20. doi: 10.1128/mBio.01781-20 (PMC7512550; doi:10.1128/mBio.01781-20)
Supplement: FIG S1 [file mBio.01781-20-sf001.pdf]

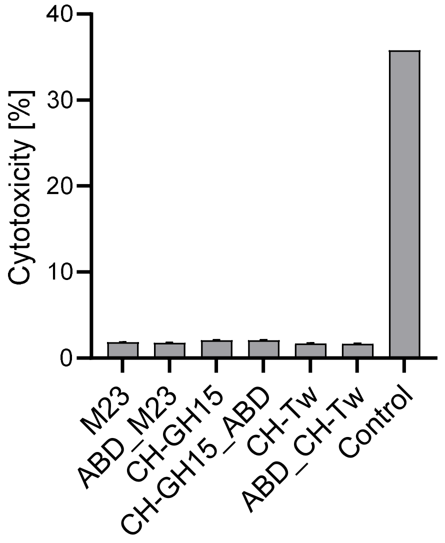

Figure S1. Cytotoxicity of parental and ABD-fused PGHs in MG-63 cells after 22 h. Cells were incubated with parental or ABD-fused constructs for 22 h, and LDH levels were measured in the supernatant with the PierceTM LDH Cytotoxicity Assay. All samples were blank corrected. The control was cell culture medium spiked with LDH provided with the kit. Values represent means with standard deviation from three independent experiments.
