# Supplementary material for: Engineering of Long-Circulating Peptidoglycan Hydrolases Enables Efficient Treatment of Systemic Staphylococcus aureus Infection
Source: mBio. 2020 Sep 22;11(5):e01781-20. doi: 10.1128/mBio.01781-20 (PMC7512550; doi:10.1128/mBio.01781-20)
Supplement: FIG S2 [file mBio.01781-20-sf002.pdf]

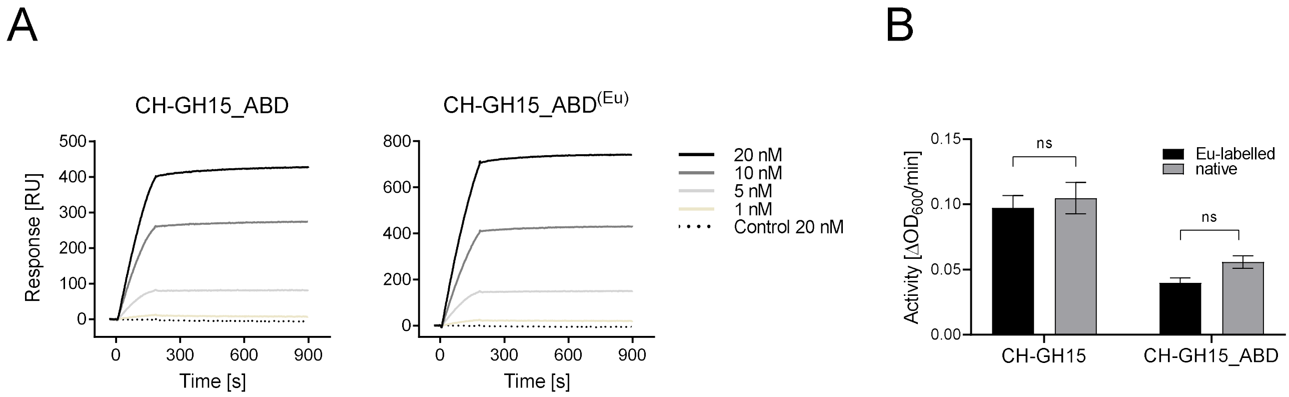

Figure S2. Influence of conjugated europium label (DTBTA-Eu^{3+}) on serum albumin binding and lytic activity of PGHs. A. Binding of native and Eu-labelled ABD-fused CH-GH15 (CH-GH15_ABD and CH-GH15_ABD$^{(EU)}$) to immobilized mouse serum albumin (MSA), as determined by SPR. Association was measured for 180 s and dissociation for 720 s at a constant flow rate of 10 µl/min. Injections of native (CH-GH15) and Eu-labelled (CH-GH15$^{(Eu)}$) parental enzymes at a concentration of 20 nM served as controls. B. Analysis of *S. aureus* lysis by native and Eu-labelled PGHs in TRAs. Enzymes at a concentration of 50 nM were mixed with *S. aureus* Newman cells, and the reduction in optical density at 600 nm was monitored over time. Error bars represent standard error of the mean from four individual experiments. ns, non-significant.
