# Supplementary material for: Engineering of Long-Circulating Peptidoglycan Hydrolases Enables Efficient Treatment of Systemic Staphylococcus aureus Infection
Source: mBio. 2020 Sep 22;11(5):e01781-20. doi: 10.1128/mBio.01781-20 (PMC7512550; doi:10.1128/mBio.01781-20)
Supplement: FIG S3 [file mBio.01781-20-sf003.pdf]

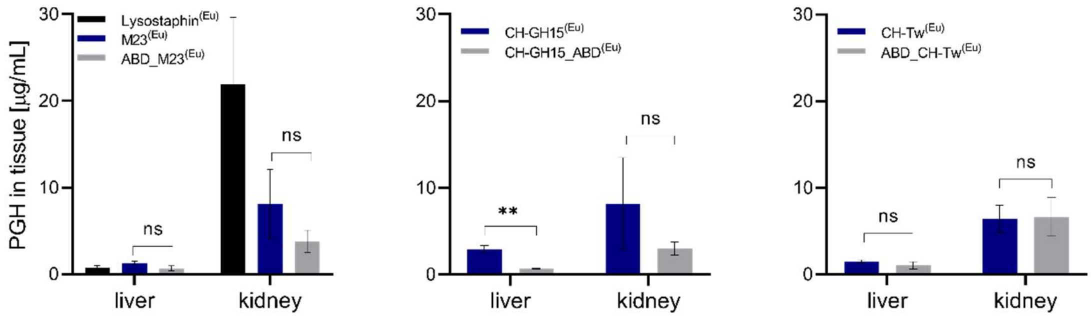

Figure S3. Biodistribution of europium-labelled PGHs in murine livers and kidneys. Eu-labelled PGHs were injected into the mice via the tail vein (5 mg/kg body weight). After 168 h, mice were sacrificed and livers and kidneys were collected and homogenized. The concentration of PGHs in the organs was determined by TRF. Error bars represent standard errors of the mean from two (CH-Tw$^{(Eu)}$) or three (all other enzymes) individual experiments. Asterisks indicate statistical significance (**, $P < 0.01$; ns, non-significant, $P > 0.05$).
