# Supplementary material for: Engineering of Long-Circulating Peptidoglycan Hydrolases Enables Efficient Treatment of Systemic Staphylococcus aureus Infection
Source: mBio. 2020 Sep 22;11(5):e01781-20. doi: 10.1128/mBio.01781-20 (PMC7512550; doi:10.1128/mBio.01781-20)
Supplement: TABLE S3 [file mBio.01781-20-st003.pdf]

Table S3. Bacterial strains used in this study.

Strain	AB^{R} [1)]	Origin	Application
E. coli BL21 Gold (DE3)	Tet^{R}	Stratagene	Cloning, expression
ClearColi® BL21 (DE3)	-	Lucigen	Endotoxin-free expression
E.coli Sure	Tet^{R}, Kan^{R}, Cam^{r}	Stratagene	Protein expression
E. coli XL1Blue MRF’	Tet^{R}	Stratagene	Protein expression
S. aureus SA113	MSSA	(Reference 1)[2)]	Activity assays
S. aureus Newman	MSSA	(Reference 2)[3)]	Activity assays
S. aureus Cowan	MSSA	(Reference 3)[4)]	Activity assays
S. aureus USA300 JE-2	MRSA	(Reference 4) [5)]	Activity assays
S. aureus ZH123	MRSA	Clinical isolate[6)]	Activity assays
S. aureus ZH313	MRSA	Clinical isolate[6)]	Activity assays
S. aureus ZH133	MRSA	Clinical isolate[6)]	Activity assays

[1)] Antibiotic resistances: - no resistances, Tet^{R}: tetracycline resistant, Kan^{R}: kanamycin resistant, Cam^{r}: chloramphenicol resistant, MSSA: methicillin susceptible *S. aureus*, MRSA: methicillin resistant *S. aureus*

[2)] Andreas Peschel, University of Tübingen, Germany

[3)] ATCC 25904

[4)] ATCC 12598

[5)] NR-46543, Network on Antimicrobial Resistance in *Staphylococcus aureus* (NARSA)

[6)] Brigitte Berger-Bächi, Institute of Medical Microbiology, University of Zurich, Switzerland

References

1. Iordanescu S, Surdeanu M. 1976. Two restriction and modification systems in *Staphylococcus aureus* NCTC8325. J Gen Microbiol 96:277-81.
2. Duthie ES, Lorenz LL. 1952. Staphylococcal coagulase; mode of action and antigenicity. J Gen Microbiol 6:95-107.
3. Bohacek J, Kocur M, Martinec T. 1971. Deoxyribonucleic acid base composition of serotype strains of *Staphylococcus aureus*. J Gen Microbiol 68:109-13.
4. Fey PD, Endres JL, Yajjala VK, Widhelm TJ, Boissy RJ, Bose JL, Bayles KW. 2013. A genetic resource for rapid and comprehensive phenotype screening of nonessential *Staphylococcus aureus* genes. mBio 4:e00537-12.
